# Industrial Acetogenic Biocatalysts: A Comparative Metabolic and Genomic Analysis

**DOI:** 10.3389/fmicb.2016.01036

**Published:** 2016-07-07

**Authors:** Frank R. Bengelsdorf, Anja Poehlein, Sonja Linder, Catarina Erz, Tim Hummel, Sabrina Hoffmeister, Rolf Daniel, Peter Dürre

**Affiliations:** ^1^Institut für Mikrobiologie und Biotechnologie, Universität UlmUlm, Germany; ^2^Genomic and Applied Microbiology and Göttingen Genomics Laboratory, Georg-August University GöttingenGöttingen, Germany

**Keywords:** synthesis gas, syngas fermentation, Wood-Ljungdahl pathway, *Clostridium ljungdahlii*, C. autoethanogenum, *C. ragsdalei*, *C. coskatii*, metabolic engineering

## Abstract

Synthesis gas (syngas) fermentation by anaerobic acetogenic bacteria employing the Wood–Ljungdahl pathway is a bioprocess for production of biofuels and biocommodities. The major fermentation products of the most relevant biocatalytic strains (*Clostridium ljungdahlii, C. autoethanogenum, C. ragsdalei*, and *C. coskatii*) are acetic acid and ethanol. A comparative metabolic and genomic analysis using the mentioned biocatalysts might offer targets for metabolic engineering and thus improve the production of compounds apart from ethanol. Autotrophic growth and product formation of the four wild type (WT) strains were compared in uncontrolled batch experiments. The genomes of *C. ragsdalei* and *C. coskatii* were sequenced and the genome sequences of all four biocatalytic strains analyzed in comparative manner. Growth and product spectra (acetate, ethanol, 2,3-butanediol) of *C. autoethanogenum, C. ljungdahlii*, and *C. ragsdalei* were rather similar. In contrast, *C. coskatii* produced significantly less ethanol and its genome sequence lacks two genes encoding aldehyde:ferredoxin oxidoreductases (AOR). Comparative genome sequence analysis of the four WT strains revealed high average nucleotide identity (ANI) of *C. ljungdahlii* and *C. autoethanogenum* (99.3%) and *C. coskatii* (98.3%). In contrast, *C. ljungdahlii* WT and *C. ragsdalei* WT showed an ANI-based similarity of only 95.8%. Additionally, recombinant *C. ljungdahlii* strains were constructed that harbor an artificial acetone synthesis operon (ASO) consisting of the following genes: *adc, ctfA, ctfB*, and *thlA* (encoding acetoacetate decarboxylase, acetoacetyl-CoA:acetate/butyrate:CoA-transferase subunits A and B, and thiolase) under the control of *thlA* promoter (P*_thlA_*) from *C. acetobutylicum* or native *pta-ack* promoter (P*_pta-ack_*) from *C. ljungdahlii*. Respective recombinant strains produced 2-propanol rather than acetone, due to the presence of a NADPH-dependent primary-secondary alcohol dehydrogenase that converts acetone to 2-propanol. Furthermore, the ClosTron^TM^ system was used to construct an *adhE1* integration mutant. These results provide extensive insights into genetic features of industrially relevant bacterial biocatalysts and expand the toolbox for metabolic engineering of acetogenic bacteria able to ferment syngas.

## Introduction

Autotrophic acetogens are able to reduce carbon monoxide (CO) and/or carbon dioxide (CO_2_) using hydrogen (H_2_) as energy source and produce acetic acid via the Wood–Ljungdahl pathway. Moreover, respective anaerobic bacteria (biocatalysts) can utilize synthesis gas (syngas), a mixture of mostly H_2_, CO, as well as CO_2_, and convert these gasses into fuels or chemicals (Daniell et al., 2012; Liew et al., 2016b). Syngas fermentation using defined biocatalysts results in a variety of products such as acetic acid, ethanol, 2,3-butanediol, butyric acid, butanol, and hexanol (Henstra et al., 2007; Phillips et al., 2015). The biochemistry of the Wood–Ljungdahl pathway is described in detail in a number of recent articles and reviews (Mock et al., 2014, 2015; Schuchmann and Müller, 2014; Diender et al., 2015). The most prominent autotrophic acetogenic biocatalysts are *C. ljungdahlii, C. autoethanogenum, C. ragsdalei*, and *C. coskatii*. These acetogenic bacteria are phylogenetically closely related (Bengelsdorf et al., 2013). They share very high identities regarding their 16S rRNA gene sequences (99–100%) and are therefore nearly indistinguishable.

Respective strains or required metabolic pathways for product formation are either protected by patents or patents are filed for approval by companies such as Coskata Inc. (Synata Bio), INEOS Bio, or LanzaTech. These international companies operate the syngas fermentation process using primarily the mentioned biocatalysts for production of biofuels and biocommodities from various gaseous feedstocks (Daniell et al., 2012). Recently, LanzaTech, ArcelorMittal, and Primetals Technologies announced a partnership to construct an industrial-scale biofuel production facility in Ghent, Belgium (LanzaTech, 2015). In September 2014, INEOS Bio stated that their “Vero Beach” facility completed a major turn-around that included upgrades to the technology (INEOS Bio, 2014). Since then, no further operational updates were released. However, a presentation published by the U.S. Department of Energy indicates that the fermentation process is impaired by hydrogen cyanide (HCN), which presumably originates during biomass gasification and is toxic for the used biocatalyst. In December 2014, the process was shut down for HCN scrubber installation (Russel, 2015). Since 2013, Coskata Inc. is not launching any news and the corresponding website is down. However, Coskata’s technology was recently transferred to a new company named Synata Bio which starts with a $10 million investment (Lane, 2016).

The bacterial names *C. autoethanogenum, C. ragsdalei*, and *C. coskatii* are not yet accepted and therefore not present in the ‘Approved Lists of Bacterial Names’ published by the journal Int J Syst Bacteriol/Int J Syst Evol Microbiol (Euzéby^1^). Only *C. ljungdahlii* is a validly described bacterial species (Tanner et al., 1993). *C. ljungdahlii* is an anaerobic, rod-shaped, Gram^+^, chemolithotrophic, motile, spore-forming, and mesophilic bacterium, which was isolated from chicken yard waste (Tanner et al., 1993). Abrini et al. (1994) published the description of *C. autoethanogenum* just a few months later and presented a very similar bacterium isolated from rabbit feces. *C. ragsdalei* is described in a patent by Huhnke et al. (2008), and the document points out characteristics that distinguish *C. ragsdalei* from *C. autoethanogenum* and *C. ljungdahlii.* Similarly, *C. coskatii* is also described in a patent (Zahn and Saxena, 2011), and the authors differentiate the strain from *C. ragsdalei, C. ljungdahlii*, and *C. autoethanogenum.* Later the three strains were studied as potential biocatalysts for ethanol and 2,3-butanediol production in numerous studies (for reviews see Bengelsdorf et al., 2013; Devarapalli and Atiyeh, 2015; Liew et al., 2016b).

Köpke et al. (2010) presented the first recombinant strain of *C. ljungdahlii* and reported the production of butanol using a metabolic engineering approach. An improved method to accomplish genetic manipulation in *C. ljungdahlii* presented by Leang et al. (2013) promotes this acetogen as a chassis for production of biocommodities. Elimination of side products, especially acetate or ethanol, would be beneficial for an efficient production of bulk chemicals (e.g., acetone or butanol) from acetyl-CoA. Inhibition of ethanol production improved acetate synthesis of recombinant *C. ljungdahlii* cells that were grown using fructose as substrate (Leang et al., 2013). Recently, a Cre-lox system for recycling of genes providing antibiotic resistance was successfully constructed for *C. ljungdahlii* (Ueki et al., 2014). Furthermore, an effective lactose-inducible promoter system (Hartman et al., 2010) was applied in *C. ljungdahlii* (Banerjee et al., 2014) allowing controlled gene expression. Moreover, acetone and butyrate formation was reported in *C. ljungdahlii* by expression of heterologous genes obtained from *C. acetobutylicum* (Banerjee et al., 2014; Ueki et al., 2014). The ClosTron^TM^ protocol was also successfully applied to construct different mutant strains of *C. autoethanogenum* (Liew et al., 2016a; Marcellin et al., 2016).

**FIGURE 1:**
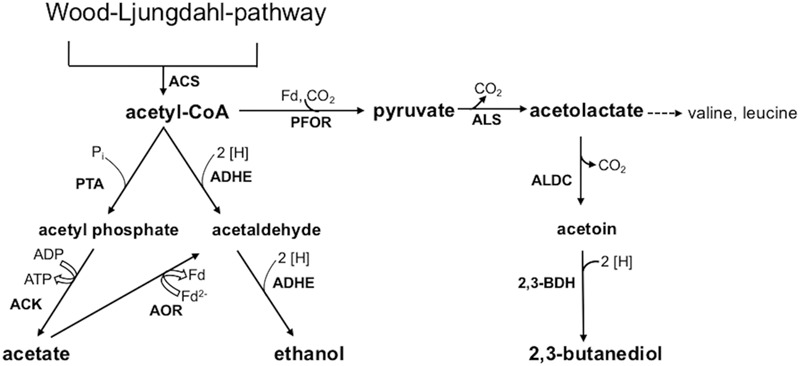
**Acetyl-CoA conversion to acetic acid, ethanol, and 2,3-butanediol.** Abbreviations: 2 [H], reducing equivalents (either NADH or NADPH); 2,3-BDH, 2,3-butanediol dehydrogenase; ACK, acetate kinase; ADHE, bifunctional aldehyde/alcohol dehydrogenase; AOR, aldehyde:ferredoxin oxidoreductase; ALDC, acetolactate decarboxylase; ALS, acetolactate synthase; Fd, ferredoxin; PFOR, pyruvate:ferredoxin oxidoreductase; PTA, phosphotransacetylase.

This study aimed at a comparative metabolic and genomic analysis of the industrial acetogenic biocatalysts *C. ljungdahlii, C. autoethanogenum, C. ragsdalei*, and *C. coskatii.* Autotrophic growth, product formation, and genomic varieties of the strains were analyzed with special emphasis regarding ethanol formation. Two different pathways are known for ethanol formation from acetyl-CoA (**Figure 1**). One pathway is a two-step reduction via acetaldehyde, which is further reduced to ethanol by a bifunctional aldehyde/alcohol dehydrogenase (ADHE). The other pathway is the conversion of the acetate to acetaldehyde by acetaldehyde:ferredoxin oxidoreductase (AOR) and further reduction to ethanol by an alcohol dehydrogenase (Abubackar et al., 2016). In order to verify the significance of one or the other pathway, the relevant genes in the genome sequences from the biocatalytic strains were compared. Furthermore, the ClosTron^TM^ system was used to study the impact of *adhE1* inactivation in a respective *C. ljungdahlii* mutant strain.

## Materials and Methods

### Bacterial Strains

*Clostridium autoethanogenum* DSM 10061, *C. ljungdahlii* DSM 13582, and *C. ragsdalei* DSM 15248 were obtained from the DSMZ (Deutsche Sammlung von Mikroorganismen und Zellkulturen GmbH, Brunswick, Germany). *C. coskatii* ATCC PTA-10522, “PS02” was ordered from the American Type Culture Collection (ATCC) Manassas, VA 20110, USA. The organisms were cultivated under strictly anaerobic conditions in modified Tanner medium (Tanner, 2007) at 37°C. *Escherichia coli* XL1-Blue MRF’ (Stratagene GmbH, Heidelberg, Germany) was used for cloning experiments. *E. coli* strains were grown aerobically at 37°C in Luria-Bertani (LB) medium (Green and Sambrook, 2012). *E. coli* strains were maintained in LB medium supplemented with 25% (v/v) glycerol at -80°C.

### Growth Conditions

Heterotrophic growth was performed with 40 mM fructose under an atmosphere of N_2_ + CO_2_ (80% + 20%), while synthesis gas (50% CO, 45% H_2_, 5% CO_2_,) was used for autotrophic growth at a pressure of 100 kPa. The modified Tanner medium (Tanner mod. medium) based on medium ATCC 1754 (Tanner, 2007) was slightly modified regarding concentrations of various components. Tanner mod. medium components (per L): 2-(*N*-morpholino) ethanesulfonic acid (MES) 20.0 g, yeast extract 0.5 g, mineral solution 25.0 mL, trace element solution 10 mL, vitamin solution 10 mL, resazurin 0.5 mg, cysteine-HCl × H_2_O 1 g. Mineral solution (per 500 mL): NaCl 40 g, NH_4_Cl 50 g, KCl 5 g, KH_2_PO_4_ 5 g, MgSO_4_ × 7 H_2_O 10 g, CaCl_2_ × 2 H_2_O 2 g. Vitamin solution (per L): pyridoxine-HCl 10 mg, thiamine-HCl × 2 H_2_O 5 mg, riboflavine 5 mg, D-Ca-pantothenate 5 mg, lipoic acid 5 mg, p-aminobenzoic acid 5 mg, nicotinic acid 5 mg, vitamin B_12_ 5 mg, biotin 2 mg, MESNA (sodium-2-mercaptoethansulfonate) 10 mg, folic acid 2 mg. For the trace element solution, it was important to dissolve first the nitrilotriacetic acid and to adjust the pH to 6.5 with KOH. Then, the other components were added, and the final pH was adjusted to 5.9 (with KOH). Trace element solution (per L): nitrilotriacetic acid 2 g, MnSO_4_ × H_2_O 1 g, Fe(NH_4_) (SO_4_)_2_ × 6 H_2_O 0.8 g, CoCl_2_ 0.2 g, ZnSO_4_ × 7 H_2_O 1 g, CuCl_2_ 0.02 g, NiCl_2_ × 6 H_2_O 0.2 g, Na_2_MoO_4_ × 2 H_2_O 0.02 g, Na_2_SeO_3_ × 5 H_2_O 0.1 g, Na_2_WO_4_ 0.2 g. Fructose (40 mM) for heterotrophic growth was added from a sterile anaerobic stock solution.

Heterotrophic growth was carried out in 50 mL medium, using 125-mL infusion flasks (Thermo Fisher Scientific) with butyl rubber stoppers. Autotrophic growth experiments were performed with 100 mL medium in 1000-mL flasks with butyl rubber stoppers. All growth experiments were carried out in biological triplicates in an uncontrolled batch mode. Growth of cells was monitored by measuring optical density at 600 nm (OD_600__nm_), and metabolic end products were analyzed by high performance liquid chromatography (HPLC).

Thiamphenicol was dissolved in dimethylformamide and added to medium (5 μg mL^-1^) in order to select recombinant *C. ljungdahlii* strains after transformation procedure. Verified recombinant strains were maintained by lyophilization for long-term storage. *C. ljungdahlii* cells were grown on agar plates (1.5% Bacto^®^-Agar) after transformation at 37°C in an anaerobic chamber with an N_2_ + H_2_ (95% + 5%) atmosphere. YTF medium (Leang et al., 2013; 10 g L^-1^ yeast extract, 16 g L^-1^ Bacto tryptone, 4 g L^-1^ NaCl, 5 g L^-1^ fructose, 0.5 g L^-1^ L-cysteine-HCl × H_2_O, pH 6) was used to obtain colony-forming units (CFU).

### Analytical Methods

Cell growth was monitored oﬄine by measuring the optical density at 600 nm (Genesys 20, Thermo Electron, Dreieich, Germany). Two milliliter samples for subsequent analysis of product concentrations were withdrawn with a syringe, centrifuged at 21.382 × *g* at 4°C for 20 min, and supernatant was stored at -20°C.

2,3-Butanediol, acetate, acetone, ethanol, fructose, and 2-propanol were determined using an ‘Agilent 1260 Infinity Series HPLC system’ (Agilent Technologies, Böblingen, Germany) equipped with a ‘Refractive Index Detector’ operating at 35°C and a ‘Diode Array Detector.’ The ‘CS-Chromatographie organic acid column’ (CS-Chromatographie Service GmbH, Langerwehe, Germany) was kept at 60°C. 5 mM H_2_SO_4_ was used as mobile phase with a flow rate of 0.7 mL/min. After thawing, samples were centrifuged again at 21.382 × *g* for 10 min at 4°C. Twenty micro liter of supernatant were injected into the HPLC system for determination of compounds.

2,3-Butanediol, acetate, acetone, and ethanol were also determined using gas chromatograph (GC) ‘clarus 600’ (PerkinElmer, PerkinElmer, Waltham MA, USA). GC was equipped with a metal column (i ø 2 mm × 2 m) packed with Porapak P 80–100 mesh. N_2_ was the carrier gas (45.0 mL min^-1^), injector temperature was 200°C, and detector temperature was 300°C. A temperature profile was predefined: 130°C for 1 min, 5°C min**^-^**^1^ increasing steps to 165°C (constant for 7 min). Supernatant (1 mL) was acidified with 0.1 mL of 2 M HCl containing 110 mM isobutanol, which served as an internal standard. One micro liter was injected into the GC.

Acetate, acetone, ethanol, and 2-propanol were determined using GC ‘HP4/Agilent GC 6890’ (Agilent Technologies; Böblingen, Germany). GC was equipped with a capillar column (DB WAX plus, 30 m × 0.25 μm × 0.25 μm). N_2_ was the carrier gas (45 mL min^-1^), injector temperature was 230°C, and detector temperature was 250°C. A temperature profile was predefined: 60°C for 2 min, 7.5°C min**^-^**^1^ increasing steps to 150°C (constant for 8 min). Supernatant (1.5 mL) was mixed with 10 mg of 2-methoxyethyl ether (dissolved in ultrasonic bath) and served as an internal standard. One micro liter of this solution was injected into the GC.

### DNA Preparation and Genome Sequencing

Standard molecular cloning techniques were performed according to established protocols (Green and Sambrook, 2012). Genomic DNA of clostridia was isolated using ‘Epicentre MasterPure^TM^ Gram Positive DNA purification kit’ (Biozym Scientific GmbH, Hessisch Oldendorf, Germany). Plasmid DNA of *E. coli* strains were obtained by ‘Zyppy^TM^ plasmid miniprep kit’ (Hiss Diagnostics GmbH, Freiburg, Germany). DNA fragments of clostridial DNA were amplified via PCR using ‘ReproFast polymerase’ (Genaxxon, Ulm, Germany).

Genomic DNA of *C. coskatii* ATCC PTA-10522 and *C. ragsdalei* DSM 15248 was sequenced using an Illumina MiSeq system (Illumina, San Diego, CA, USA). Illumina shotgun libraries were generated from the extracted DNA according to the protocol of the manufacturer. Sequencing resulted in 2,179,216 300-bp paired end reads for *C. coskatii* and 2,179,216 300-bp for *C. ragsdalei*. Reads were trimmed using Trimmomatic 0.32 (Bolger et al., 2014) to remove sequences with quality scores lower than 20 (Illumina 1.9 encoding) and remaining adaptor sequences.

The *de novo* assembly performed with the SPAdes genome assembler software 3.5.0 (Bankevich et al., 2012) resulted in 112 contigs (>500 bp) for *C. coskatii*, in 79 contigs (>500 bp) for *C. ragsdalei* and an average coverage of 91.62-fold and 396.2-fold, respectively. Automatic gene prediction was performed by using the software tool Prodigal (Hyatt et al., 2010). Genes coding for rRNA and tRNA were identified using RNAmmer (Lagesen et al., 2007) and tRNAscan (Lowe and Eddy, 1997), respectively. The IMG-ER system (Markowitz et al., 2014) was used for automatic annotation, which was subsequently manually curated by using the Swiss-Prot, TrEMBL, and InterPro databases (Zdobnov and Apweiler, 2001). Genome sequences have been deposited at DDBJ/EMBL/GenBank under the accession numbers LROR00000000 (*C. coskatii* PTA-10522) and LROS00000000 (*C. ragsdalei* P11). The versions described in this paper are versions LROR01000000 and LROS01000000, respectively.

### Plasmids

An artificial acetone synthesis operon (ASO) developed previously (Lederle, 2010) was cloned in the pJIR750 vector system (Hoffmeister et al., 2016) and transformed in *C. ljungdahlii* cells to validate acetone formation by recombinant strains. The ASO ((shortened form ‘act’) contained genes *adc* (encoding acetoacetate decarboxylase,), *ctfA, ctfB*, (acetoacetyl CoA:acetate/ butyrate:CoA transferase subunit A and B) and *thlA* (thiolase) under control of P*thlA* promoter (promoter of the thiolase gene), representing the known acetone synthesis pathway of *C. acetobutylicum*. Furthermore, P*thlA* promoter of ASO was exchanged by promoter of genes *pta* and *ack* encoding phosphotransacetylase and acetate kinase from *C. ljungdahlii*, respectively (Hoffmeister et al., 2016). Thus, ASO was controlled by a native promoter from *C. ljungdahlii* in plasmid pJIR_act_P_*_pta-ack_* (**Table 1**). In order to disrupt the *adhE1* gene (locus tag CLJU_c16510) encoding a bifunctional aldehyde/alcohol dehydrogenase in *C. ljungdahlii*, we used the ClosTron^TM^ system (Heap et al., 2010). This system permitted a directed construction of stable mutants in *Clostridium* species using a bacterial group II intron. The plasmid that mediates an integration event into the gene *adhE1* was designed by following the instruction of the ‘Intron targeting and design tool.’ Intron target site was identified using Perutka algorithm that is implemented in the respective online tool^2^. Selected target site was between nucleotides 114 and 115 of the nucleotide sequence of *adhE1* gene (CLJU_c16510). The resulting ‘intron targeting region’ sequence (309 bp) was synthesized by the company DNA2.0 (Menlo Park, CA, USA) and cloned into the vector pMTL007C-E2. The final plasmid (pMTL007C-E2_adhE1::intron) was used for electrotransformation of *C. ljungdahlii* WT cells (**Table 1**).

**Table 1:** Plasmids used in this study.

Plasmid	Plasmid marker	Gram + replicon	Feature	Reference
pJIR750	*catP*	pIP404	Non (control)	Bannam and Rood, 1993
pJIR_act_PthlA_	*catP*	pIP404	ASO^1^ controlled by P*thlA* promoter	Hoffmeister et al., 2016
pJIR_act_Ppta-ack_	*catP*	pIP404	ASO^1^ controlled by P*pta-ack* promoter	Hoffmeister et al., 2016
pMTL007C-E2_adhE1::intron^2^	*catP*	pCB102	Mediating *adhE1* integration event	Heap et al., 2010

*^1^ASO, acetone synthesis operon.*

### Preparation of Electrocompetent *C. ljungdahlii* Cells

Electrocompetent *C. ljungdahlii* cells were prepared according to a modified protocol of Leang et al. (2013). All plastic material was placed in the anaerobic chamber the day before transformation to eliminate remaining oxygen.

About 15 h before preparation of competent cells, a mid- to late-log-phase culture was transferred to an infusion flask with 100 mL Tanner mod. medium supplemented with 40 mM DL-threonine and 40 mM fructose (OD_600nm_: 0.06). After overnight growth at 37°C, early log-phase cells (OD_600__nm_: 0.3–0.5) were harvested by centrifugation at 9,418 × *g* for 10 min at 4°C. Cells were washed twice with 50 mL anoxic SMP buffer (270 mM sucrose, 1 mM MgCl_2_, 7 mM sodium phosphate, pH 6) and suspended in 0.6 mL of the same buffer. Afterward, 120 μL anoxic anti-freezing buffer (60% DMSO and 40% SMP buffer, pH 6) were added to competent cells. These cells were stored in “cryo tubes” at -80°C for further use.

### Electrotransformation of *C. ljungdahlii*

Transformation procedure was carried out in an anaerobic chamber. Twenty five micro liter of electrocompetent *C. ljungdahlii* cells were mixed with 5 μg of plasmid DNA and transferred to a pre-cooled 0.1-cm gap electroporation cuvette (Biozym Scientific). Electric pulse was performed with 625 V, resistance of 600 Ω, and a capacitance of 25 μF using a ‘Gene-Pulser^®^ II with Pulse Controller Plus’ (Bio-Rad Laboratories GmbH, München, Germany). Afterward, cells were recovered using 0.5 mL Tanner mod. medium without antibiotic in a Hungate tube with 5 mL medium. OD_600nm_ was controlled after transformation (0.05–0.09). Regeneration was carried out overnight at 37°C. The next day, OD_600__nm_ was checked again (0.6–0.7) and 600 μL of the regenerated culture were plated on YTF agar (pH 6) with the appropriate antibiotic in an anaerobic chamber, and plates were incubated upside down at 37°C. After about 5 days, single colonies of obtained transformants were picked, and presence of the plasmid was confirmed by isolating genomic DNA and retransformation into *E. coli* XL1-Blue MRF’ and by detecting the respective plasmid via standard PCR using the following primers: pJIR750_fwd_, gataaccgtattaccgcctttg; pJIR750_rev_ gcacagatgcgtaaggag. Integration mutants were verified by PCR using primers targeting the gene *adhE1* (P_fwd_, 5′-catcaaggggtttatttgtc-3′; P_rev_, 5′-atctctctctaaaactccac-3′).

### *In Silico* Analysis

High quality genome sequences are available for *C. ljungdahlii* (Köpke et al., 2010) and *C. autoethanogenum* (Brown et al., 2014; Humphreys et al., 2015). A draft genome sequence of *C. ragsdalei* (328 contigs) is accessible using the “Integrated Microbial Genomes-Expert Review” (IMG/ER) system (Markowitz et al., 2014). A draft genome sequence of *C. coskatii* was recently listed by Martin et al. (2015), but unfortunately, the authors deposited only raw data (SRR1970390) and not an annotated genome sequence at the NCBI (National Center for Biotechnology Information) database. Therefore, all subsequent analyses were performed using the genome sequences listed in **Table 2**. Genome sequences of *C. ljungdahlii* DSM 13582, *C. autoethanogenum* DSM 10061, *C. ragsdalei* DSM 15248, and *C. coskatii* ATCC PTA-10522 (**Table 2**) were analyzed using ‘IMG/ER system’ (Markowitz et al., 2014) provided by the ‘DOE Joint Genome Institute’ (Walnut Creek, CA, USA). Orthologous genes (orthologs) among genome sequences were identified using Proteinortho version 4.26 (default specification: blast = blastp v2.2.24, *E*-value = 1e-10, alg.-conn. = 0.1, coverage = 0.5, percent_identity = 50, adaptive_similarity = 0.95, inc_pairs = 1, inc_singles = 1, selfblast = 1, unambiguous = 0) (Lechner et al., 2011). The respective excel file is available in the supplement (**Supplementary Table S1**). Detailed gene analysis and comparison was done using ‘CLC Workbench 7’ (CLC Bio, a QIAGEN Company, Boston, MA, USA). Gene sequences encoding alcohol dehydrogenases were derived from respective genome sequences and a multiple sequence alignment was calculated using MAFFT (Katoh and Toh, 2008). Phylogenetic tree was reconstructed with the program MrBayes v3.2.5 (Ronquist et al., 2012).

**Table 2:** Features of genomes of acetogenic bacteria.

Strain, designation, culture collection ID	NCBI Accession no.	Genome size (bp)	Gene count	CRISPR count	GC [%]	Contigs
*C. ljungdahlii*, “PETC,” DSM 13528^b^	CP001666	4,630,065	4,184	1	31	1
*C. autoethanogenum*, “JA1-1,” DSM 10061^a^	CP006763	4,352,205	4,024	4	31	1
*C. ragsdalei*, “P11,” DSM 15248^c^	LROS01000000	4,424,992	3,946	1	31	79
*C. coskatii*, “PS02,” ATCC PTA-10522^c^	LROR01000000	4,538,837	4,211	4	31	112

*^a^Brown et al., 2014; ^b^Köpke et al., 2010; ^c^this study.*

## Results

### Growth and Metabolic Profiles

**FIGURE 2:**
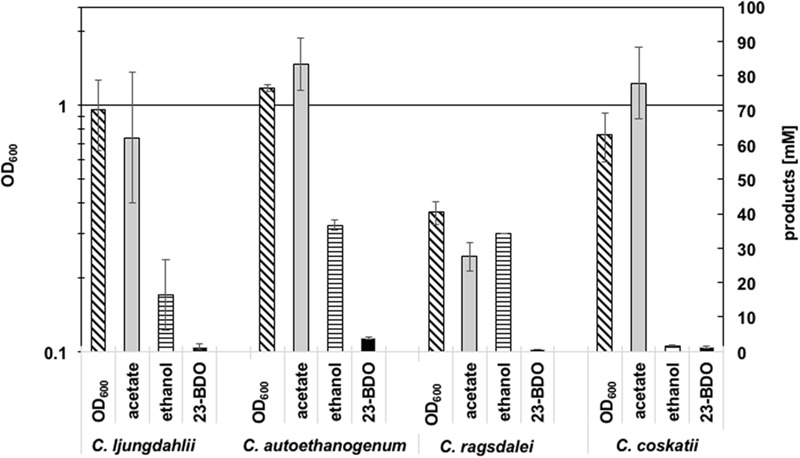
**Summary of amounts of biomass (OD_600_), acetate (mM), ethanol (mM), and 2,3-butanediol (mM) (23-BDO).** Error bars show standard deviations.

Growth and metabolic profiles of the four wild type (WT) strains *C. ljungdahlii* DSM 13582, *C. autoethanogenum* DSM 10061, *C. ragsdalei* DSM 15248, and *C. coskatii* ATCC PTA-10522, growing on syngas as substrate were compared in uncontrolled batch experiments (**Figure 2**). Detailed growth and product pattern curves are presented in the supplement (**Supplementary Figure S1**, **Supplementary Table S2**). In general, production of acetate, ethanol, and 2,3-butanediol correlated with the exponential growth phase of the strains (**Supplementary Figure S1**). No significant product formation was monitored in the stationary growth phase, except for *C. coskatii*, which further produced acetate. *C. autoethanogenum* reached the highest amounts of acetate, ethanol, 2,3-butanediol, and biomass (OD_600_) compared to the other tested strains (after 530 h). Although *C. ragsdalei* reached the lowest optical density (OD_600_ 0.43), the strain produced as much ethanol (34.7 mM ± 8.7) as *C. autoethanogenum*, but showing an acetate to ethanol ratio of 1:1 (instead of 2:1 in case of *C. autoethanogenum*). The strain *C. coskatii* produced significantly less ethanol under anaerobic conditions (1.4 mM ± 0.3) than the other three strains. Instead, this strain showed a continuous acetate production as well as a continuous increase of biomass. Growth and metabolic profile of *C. ljungdahlii* did not differ significantly compared to that of *C. autoethanogenum* and *C. ragsdalei*, but were characterized by a high variability as indicated by the standard deviation values (**Supplementary Figure S1**).

### Genome-Wide Comparison of Biocatalytic Strains

**FIGURE 3:**
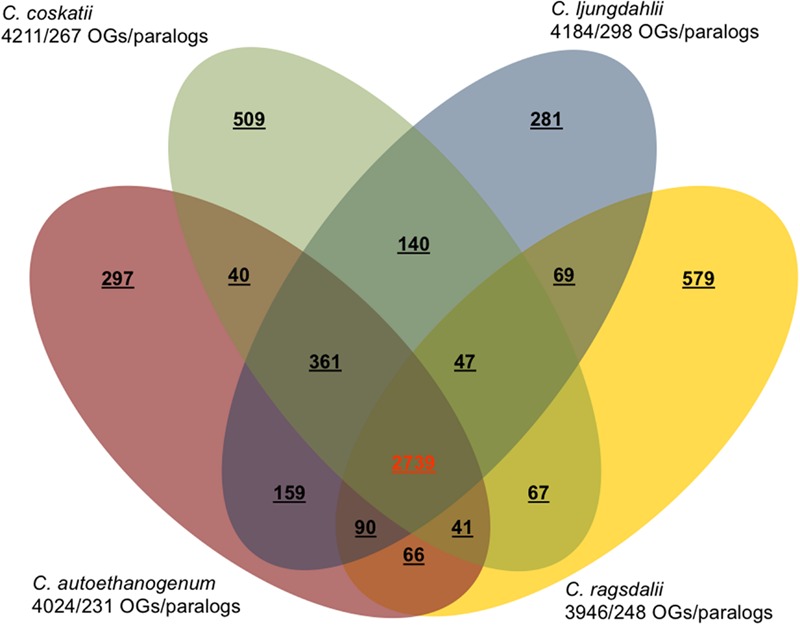
**Pan/core genome analysis of acetogenic biocatalysts.** Venn diagram showing the numbers of orthologous genes (OGs) in the core, dispensable, and specific genome of each strain (*C. ljungdahlii, C. autoethanogenum, C. ragsdalei*, and *C. coskatii*). Ortholog detection was done with the Proteinortho software (blastp) with a similarity cut-off of 50% and an *E*-value of 1e^-10^. The total number of genes and paralogs, respectively, are depicted under the corresponding species name. Open reading frames that were classified as pseudogenes, were not included in this analysis.

**FIGURE 4:**
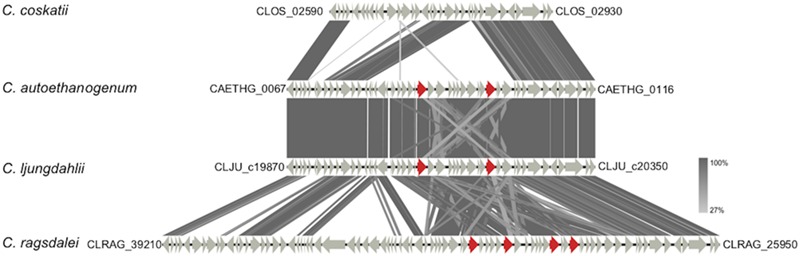
**TBLASTx comparison of gene clusters containing genes coding for aldehyde: ferredoxin oxidoreductases (AORs).** Genes encoding AORs are marked in red (locus tags are provided in the text). For the comparison, an *E*-value cut-off of 1e^-10^ was used, and visualization of the gene clusters was done using the program Easyfig (Sullivan et al., 2011). Locus tags shown in the figure indicate start and end of the displayed gene clusters. In case of *C. ragsdalei* the displayed gene cluster is scattered over different contigs.

The major characteristics of the genomes of *C. ljungdahlii, C. autoethanogenum, C. ragsdalei*, and *C. coskatii*, are listed in **Table 2**. The genome sequencing of *C. coskatii* and *C. ragsdalei* was performed in this study. The 16S rRNA gene sequences of the four bacterial strains show very high similarities (99–100%) and are therefore not suitable to distinguish the strains from each other. An average nucleotide identity (ANI) analysis also showed high similarities between the genomes of *C. ljungdahlii* and *C. autoethanogenum* (99.3%) as well as *C. ljungdahlii* and *C. coskatii* (98.3%). However, *C. ljungdahlii* and *C. ragsdalei* showed an ANI similarity of only 95.8%. Pan/core genome analysis based on orthologous genes (OGs) detection performed for the four acetogenic bacteria is shown in **Figure 3**. The pan genome of the four organisms consists of 5,485 OGs, including core genome, dispensable genome (OGs shared by at least 2 genomes) and genome specific OGs (singletons). The core genome consists of 2,739 OGs and represents 50% of all proteins present in each genome. Only, 8.3% of the 2,739 OGs in the core genome are hypothetical proteins. Moreover, *C. ljungdahlii, C. autoethanogenum*, and *C. coskatii* share 3,100 protein sequences which corresponds to 56.6% of all present proteins in the pan genome. However, *C. ragsdalei* harbors the highest number of singletons (579), followed by *C. coskatii*, which harbors 509 singletons. *C. ljundahlii* and *C. autoethanogenum* have with 279 and 281 OGs, respectively, a similar number of singletons. About 51% of singletons in the specific genome of all strains account for hypothetical proteins. Further abundant genes in specific genome encode transporters, phage associated proteins, and CRISPR (subtype 1B) associated proteins. **Figure 4** shows a circular representation of the genome comparison of the four biocatalytic strains. For *C. ljungdahlii* the genes encoded by the leading and the lagging strand (outer circles 1 and 2) are marked in COG colors in the artificial chromosome map. The next circle (3) presents the genes of the core genome of all biocatalytic strains. Moreover, the presence of OGs is indicated for the genomes of *C. autoethanogenum, C. ragsdalei*, and *C. coskatii* (circle 4–6) in comparison to the *C. ljungdahlii* genome. The yellow colored regions indicate low similarity, whereas red colored regions indicate high similarity (see color code, **Figure 4**). This result is in accordance with the high ANI values mentioned before. The most notable regions of differences in the genome of *C. ljungdahlii* compared to the other strains are marked by the letters a to f. The labeled regions (a to h) harbor genes encoding proteins of: prophagic regions (a and b), a number of hypothetical proteins (c), another prophage region (d), hypothetical proteins, methyltransferases, ABC transporters (e), glycine reductase complex as well as amino acid transporters (f), and finally another two prophagic regions (g and h). Further regions of differences contain genes encoding mainly hypothetical proteins. The two innermost plots represent the GC content and the GC skew (circle 7–8).

The presence or absence of genes encoding alcohol dehydrogenases (e. g. responsible for the formation of, e.g., 2,3-butanediol, ethanol, 2-propanol, and 1,3-propanediol) were analyzed in all genome sequences (**Supplementary Figure S2**). 20 genes encoding alcohol dehydrogenases (including paralogous genes) are present in the genome of *C. ljungdahlii, C. autoethanogenum*, and *C. coskatii*, whereas *C. ragsdalei* contains only 16 respective genes. In the genome of *C. ragsdalei*, the OGs for CLJU_c19540, CLJU_c11560, CLJU_c25840, and CLJU_c16150 of *C. ljungdahlii* are missing. Different alcohol dehydrogenases were previously assigned into clusters of orthologous groups of proteins (COGs). Analyzed genomes contained alcohol dehydrogenases classified as COG1062 (FrmA), COG1063 (Tdh), COG1454 (EutG), COG1979 (YqhD) and a so far unknown COG (**Supplementary Figure S2**). Most of the alcohol dehydrogenases are iron-containing alcohol dehydrogenases (COG1454, COG1979, and the unknown COG), which are assigned to one superfamily (cl02872). A number of alcohol dehydrogenases were classified as threonine dehydrogenases, or related Zn-dependent dehydrogenases (COG1063). The remaining ones were just assigned as Zn-dependent alcohol dehydrogenases (COG1062).

**FIGURE 5:**
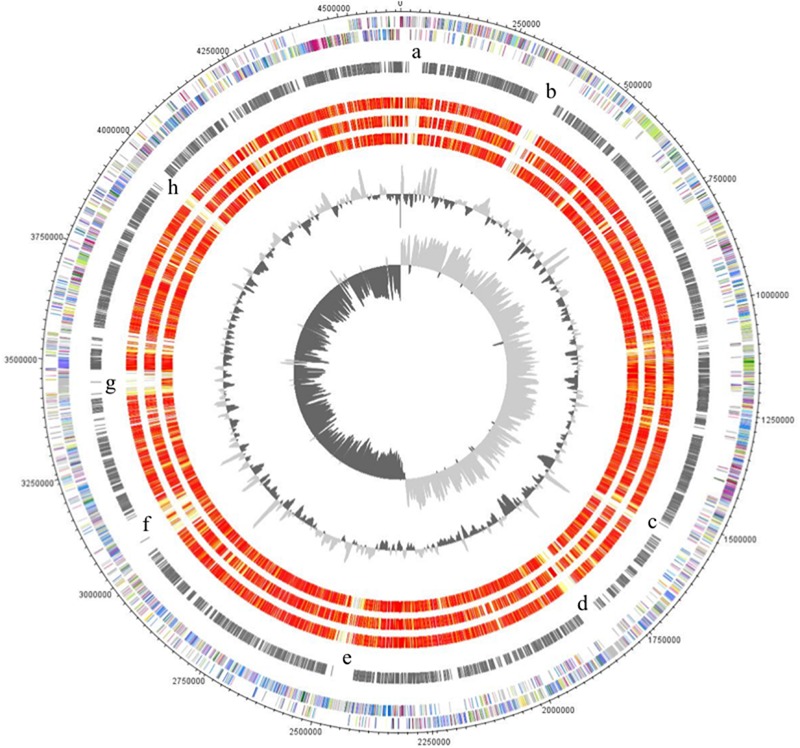
**Circular representation of the genome comparison of *C. ljungdahlii* with other biocatalytic strains.** The genes encoded by the leading and the lagging strand (outer circles 1 and 2) of *C. ljungdahlii* are marked in COG colors in the artificial chromosome map. The genes present in the core genome of all biocatalytic strains are shown in circle 3. The presence of orthologous genes [red, high similarity; orange, medium similarity; yellow, low similarity (see color code below)] is indicated for the genomes of *C. autoethanogenum, C. ragsdalei*, and *C. coskatii* (circle 4–6) in comparison to the *C. ljungdahlii* genome. The two innermost plots represent the GC content and the GC skew (circle 7–8). Visualization was done using Proteinortho results (**Supplementary Table S1**) and DNAPoltter (Carver et al., 2009). COG categories of the genes were extracted from IMG database entries of *C. ljungdahlii.* Color code according to *E*-values of the blastp analysis performed using Proteinortho 4.26. Gray, 1e^-20^ to 1; light yellow, 1e^-21^ to 1e^-50^; gold, 1e^-51^ to 1e^-90^; light orange, 1e^-91^ to 1e^-100^; orange, 1e^-101^ to 1e^-120^; red, >1e^-120^.

Regarding ethanol formation, it turned out that the strain *C. coskatii* lacks a cluster of genes including the genes encoding aldehyde:ferredoxin oxidoreductases (AORs) (**Figure 5**). The strains *C. ljungdahlii* and *C. autoethanogenum* carry two gene copies encoding AORs [CLJU_c20110 (*aor1*), CLJU_c20210 (*aor2*), CAETHG_0092 (*aor1*) and CAETHG_0102 (*aor2*)], whereas the strain *C. ragsdalei* has four gene copies [CLRAG_29620 and CLRAG_29650 (*aor1*) as well as CLRAG_29560 and CLRAG_29710 (*aor2*)]) coding for the corresponding enzymes (**Figure 5**).

### Verification of Recombinant *C. ljungdahlii* Strains

**FIGURE 6:**
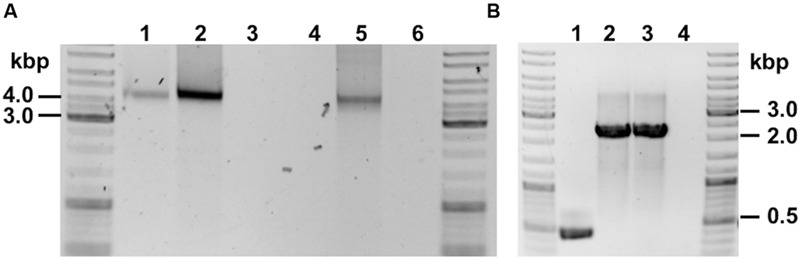
**PCR verification of recombinant *C. ljungdahlii* strains. (A)** Detection of plasmid DNA within gDNA of: 1, *C. ljungdahlii* [pJIR_act_P_*_thlA_*]; 2, *C. ljungdahlii* [pJIR_act_Ppta-ack_]. Controls: 3, *C. ljungdahlii* WT; 4, negative control (water); 5, *E. coli* [pJIR_act_PthlA_], 6, negative control (water). **(B)** Detection of integration cassette using gDNA as template of: 1, *C. ljungdahlii* WT (negative control); 2 and 3, two separate *C. ljungdahlii* [*adhE1*::intron] strains; 4, negative control (water).

Genomic DNA (gDNA) extracts of recombinant strains harboring the ASO (*C. ljungdahlii* [pJIR_act_P_*_thlA_*] and *C. ljungdahlii* [pJIR_act_P_*_pta-ack_*]) as well as the control strain (*C. ljungdahlii* [pJIR750]) were used to target respective plasmids by PCR. Plasmid DNA was amplified and the obtained DNA fragments had the expected lengths (**Figure 6A**). The respective DNA fragments were verified by Sanger sequencing.

In order to test the ClosTron^TM^ system and to obtain an *adhE1* integration mutant, cells of *C. ljungdahlii* WT were electroporated using plasmid pMTL007C-E2_adhE1::intron. Thereafter, cells were incubated anaerobically on YTF agar plates containing 15 μg mL^-1^ thiamphenicol until colonies appeared. Ten of those colonies were inoculated in 5 mL of Tanner mod. medium in Hungate tubes using clarithromycin (5 μg mL^-1^) in order to induce the integration event. After at least three reinoculation steps, two integration mutants were verified by PCR with primers targeting the gene *adhE1* (**Figure 6B**).

### Growth and Product Pattern of Recombinant *C. ljungdahlii* Strains

**FIGURE 7:**
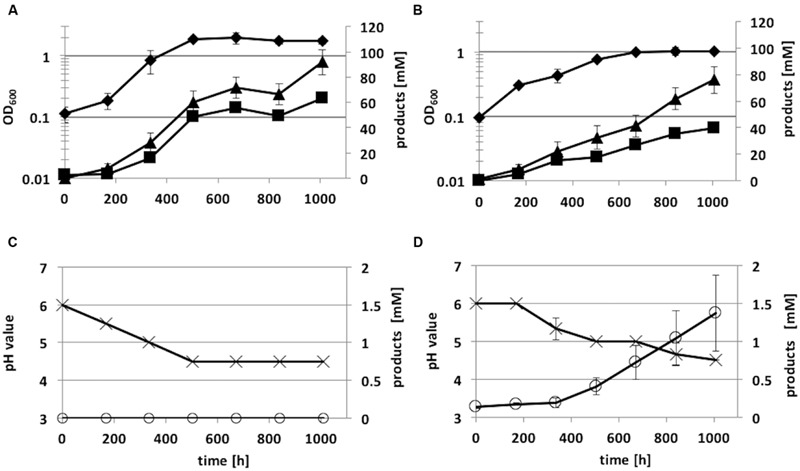
**Autotrophic growth characteristics of *C. ljungdahlii* [pJIR_act_P_*_thlA_*] **(A,C)** and *C. ljungdahlii* [pJIR_act_Ppta-ack_] **(B,D)** cultivated in 1 L Müller-Krempel bottles on syngas in 100 mL Tanner mod.** Medium in an uncontrolled batch fermentation: diamond, OD_600_; cross, pH; triangle, acetate; square, ethanol; open circle, 2-propanol. Error bars show standard deviations.

The recombinant strains *C. ljungdahlii* [pJIR_act_P_*_thlA_*] and *C. ljungdahlii* [pJIR_act_P_*_pta-ack_*] produced 2-propanol (5 ± 1 mM) rather than acetone under heterotrophic growth conditions. Thereafter, autotrophic growth of both recombinant 2-propanol production strains as well as of *C. ljungdahlii* WT and the control strain *C. ljungdahlii* [pJIR750] was monitored for 1008 h (**Figure 7**). During cultivation of cells, syngas as substrate was refilled after 336 and 772 h of incubation. The recombinant strains *C. ljungdahlii* [pJIR750] and *C. ljungdahlii* [pJIR_act_P_*_thlA_*] showed similar growth profiles compared to the WT strain indicated by similar amounts of biomass, acetate, ethanol, and 2,3-butanediol (**Table 3**). Interestingly, *C. ljungdahlii* [pJIR_act_P_*_thlA_*] was not able to produce 2-propanol under autotrophic growth conditions. Nevertheless, *C. ljungdahlii* [pJIR_act_Ppta-ack_] produced small amounts of 2-propanol (1.4 mM ± 0.5) under autotrophic growth conditions accompanied with lower biomass, acetate, ethanol, and no 2,3-butanediol production (**Table 3**). The presence of 2-propanol in the fermentation broth was verified by means of two independent analytical methods, namely HPLC and GC.

**Table 3:** Autotrophic growth characteristics of *C. ljungdahlii* WT and recombinant *C. ljungdahlii* strains during uncontrolled batch fermentation.

*C. ljungdahlii* strains	Maximum OD_600_	Acetate [mM]	Ethanol [mM]	2,3-Butanediol [mM]	2-propanol [mM]
WT^a^	1.6 ± 0.3	135.8 ± 35.5	65.2 ± 86.6	2.4 ± 1.3	Not detected
[pJIR750]^a^	1.9 ± 0.2	128 ± 6.2	63.6 ± 2.5	0.6 ± 0.2	Not detected
[pJIR_act_PthlA_]^a^	1.7 ± 0.2	91.8 ± 8.0	63.3 ± 17.4	3.8 ± 0.2	Not detected
[pJIR_act_Ppta-ack_]^a^	1.0 ± 0	75.9 ± 5.7	39.7 ± 1.7	Not detected	1.4 ± 0.5
WT^b^	1.2 ± 0.1	63.8 ± 9.2	35.2 ± 16.6	2.4 ± 1.3	Not detected
*adhE1*::intron^b^	0.9 ± 0	45.8 ± 9.8	6.9 ± 2.2	0.6 ± 0.3	Not detected

*Data represent mean values after 1008 h incubation, ± standard deviations of biological triplicates. ^a^ Syngas as substrate was refilled after 336 and 772 h of incubation. ^b^ Syngas as substrate was not refilled.*

**FIGURE 8:**
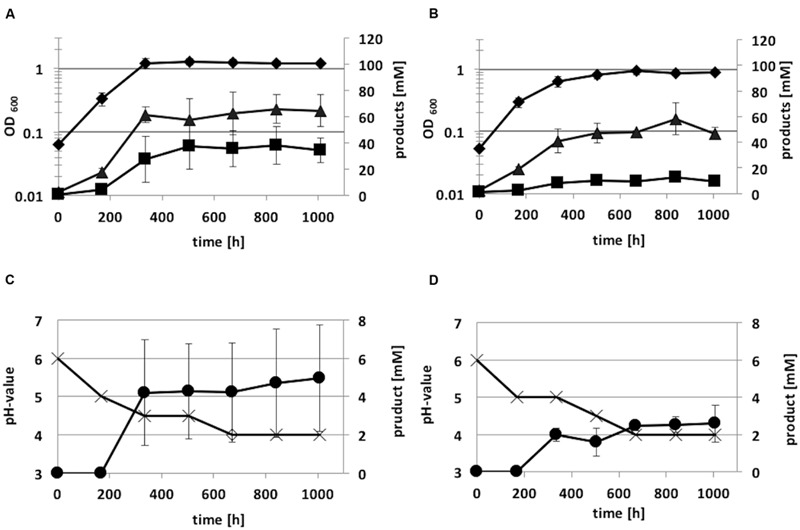
**Autotrophic growth characteristics of *C. ljungdahlii* WT **(A,C)** and *C. ljungdahlii adhE1*::intron **(B,D)** cultivated in 1 L Müller-Krempel bottles on syngas in 100 mL Tanner mod.** Medium in an uncontrolled batch fermentation: diamond, OD_600_; cross, pH; triangle, acetate; square, ethanol; circle, 2,3-butanediol. Error bars show standard deviations.

Autotrophic growth of the integration mutant *C. ljungdahlii* [*adhE1*::intron] and *C. ljungdahlii* WT was also monitored for 1008 h (**Figure 8**). Growth and acetate production of the integration mutant on syngas was not as high as that of the wild-type strain. Anyhow, the acetate:ethanol ratio of *C. ljungdahlii* [*adhE1*::intron] was 7:1, whereas *C. ljungdahlii* WT showed a lower ratio (2:1). Thus, ethanol production was reduced by a factor of five due to the integration event in the *adhE1* gene.

## Discussion

### Comparative Metabolic and Genomic Analysis

The natural ability of *C. ljungdahlii, C. autoethanogenum, C. ragsdalei, C. coskatii*, and other autotrophic acetogens to use gaseous substrates for growth and product formation enable a sustainable way to reduce green house gasses emissions that otherwise would impact earth’s climate (Dürre and Eikmanns, 2015). Autotrophic growth behavior and product spectrum (acetate, ethanol, 2,3-butanediol) of *C. ljungdahlii, C. autoethanogenum*, and *C. ragsdalei* in uncontrolled batch experiments were similar. In previous studies (Abrini et al., 1994; Köpke et al., 2011; Isom et al., 2015), acetate and ethanol were also the major metabolic end products. As shown by Isom et al. (2015), *C. ragsdalei* did not produce detectable amounts of 2,3-butanediol under the applied experimental conditions of this study, although the strain should be able to generate small amounts of 2,3-butanediol (Köpke et al., 2011). In contrast to the other strains, *C. coskatii* was the only one that produced significantly lower amounts of ethanol (1.4 ± 0.3 mM; 0.05 g L^-1^) and showed a continuous acetate production during growth. This result is in contrast to the results previously published in the patent of Zahn and Saxena (2011). According to the data in that patent, *C. coskatii* produces up to 10 g L^-1^ ethanol in batch experiments using bottles and up to 20 g L^-1^ ethanol using a synthesis gas-fed CSTR (continuous stirred tank reactor). The medium used by Zahn and Saxena (2011) was similar to the medium used in this study. Further validation of results is not feasible due to a lack of published data concerning growth and amounts of produced end products of *C. coskatii.* However, *C. coskatii* is the only investigated biocatalyst not possessing genes encoding aldehyde:ferredoxin oxidoreductases (**Figure 5**). Martin et al. (2015) also stated that the genome sequence of *C. coskatii* is lacking two AOR genes. This finding suggests ethanol formation in acetogens is linked to acetate production via AOR enzymes converting acetate into acetaldehyde, which is further converted to ethanol by alcohol dehydrogenases (Köpke et al., 2011). This is supported by the fact that *aor1* (CAETHG_0092) of *C. autoethanogenum* was highly expressed only under autotrophic and not under heterotrophic growth conditions (Marcellin et al., 2016). Under autotrophic conditions, the required reduced ferredoxin for the AOR reaction originates either from CO oxidation via the CO dehydrogenase or from H_2_ reduction via the electron-bifurcating hydrogenase. *C. coskatii* is an interesting option for recombinant production of biofuels and biocommodities, since the side product ethanol is produced only in minor amounts.

The functions of only four out of the 20 alcohol dehydrogenases encoded in the genome sequences of *C. ljungdahlii* or *C. autoethanogenum* have been investigated so far. In *C. ljungdahlii*, the genes encoding AdhE1 and AdhE2 were deleted and the respective recombinant strains produced less ethanol (Leang et al., 2013; Ueki et al., 2014). Köpke et al. (2014) showed that *C. autoethanogenum* possesses two alcohol dehydrogenases (encoded by CAETHG_0385 and CAETHG_0553) that reduce acetoin to 2,3-butanediol. First, the 2,3-butanediol dehydrogenase (2,3-Bdh; CAETHG_0385) is reducing stereospecifically *R-*acetoin to 2*R*,3*R*-butanediol. Second, a strictly NADPH-dependent primary-secondary alcohol dehydrogenase (CaADH; CAETHG_0553) equally reduces *R-*acetoin to 2*R*,3*R*-butanediol, and converts also acetone to 2-propanol (Köpke et al., 2014). Marcellin et al. (2016) investigated recently energy metabolism of *C. autoethanogenum* by a systematic approach. Under autotrophic growth conditions a specific alcohol dehydrogenase (CAETHG_1841) and during heterotrophic growth, a different alcohol dehydrogenase (CAETHG_3954) was significantly up-regulated. Any suggestions with respect to functions of those alcohol dehydrogenases were not provided (Marcellin et al., 2016). For *C. ragsdalei*, it was demonstrated that acids such as propionate, butyrate, pentanoate, and hexanoate were converted into their corresponding alcohols 1-propanol, 1-butanol, 1-pentanol, and 1-hexanol using alcohol dehydrogenases (Isom et al., 2015). Thus, detailed knowledge regarding the corresponding enzymes and genes would be helpful to construct recombinant biocatalysts that produce desired products.

Verbeke et al. (2013) performed a genome-based analysis of different *Thermoanaerobacter* species. It turned out that these species also possess a high number of alcohol dehydrogenases corresponding to the COG classes COG1063, COG1454, and COG1979. Different species of the genus *Thermoanaerobacter* are able to utilize sugar alcohols. Thus, Verbeke et al. (2013) speculated that some of the alcohol dehydrogenases have catabolic functions and do not participate in ethanol synthesis. Nevertheless, the four biocatalysts do not utilize sugar alcohols such as glycerol, mannitol, or sorbitol (Huhnke et al., 2008; Zahn and Saxena, 2011) as sole carbon sources, but possibly as co-substrate together with another carbon source (CO or fructose). Co-utilization was previously described for *C. ljungdahlii*, which metabolizes butanol only in presence of an additional carbon source (Köpke et al., 2010).

Comparative genome analysis of the four biocatalytic strains revealed high similarities between *C. ljungdahlii, C. autoethanogenum*, and *C. coskatii* (at least 98.3%) using ANI analysis. The ANI-based similarity of *C. ljungdahlii* and *C. ragsdalei* is 95.8%. The respective threshold range for species demarcation is 95–96% (Kim et al., 2014). Thus, there is still not enough evidence to prove that *C. ragsdalei* is a distinct species, although it is to some extent different from the other three strains (**Figure 3**). Approximately 50% of the proteins encoded in the individual genomes are shared by the four acetogenic biocatalysts. All shared and specific proteins are listed in **Supplementary Table S1**. For core/pan genome analysis two complete and two draft genomes were used. The use of draft genomes has nearly no effect on the size of the core or pan genome, as the gaps in the draft genomes represent repetitive regions such as rRNA clusters, transposases, or phage regions which are mainly covered by small contigs (0.5–5 kb) (Ullrich et al., 2015). Both draft genomes contain contigs larger than 0.5 kb.

All strains share the following genes or gene clusters encoding proteins or protein complexes for: Wood–Ljungdahl pathway and CO-dehydrogenase/acetyl-CoA synthetase complex (Poehlein et al., 2015), *Rnf* complex (*R*hodobacter *n*itrogen *f*ixation), ferredoxin-dependent transhydrogenase (Nfn) (Schuchmann and Müller, 2014), predicted nitrate reductase complex (Brown et al., 2014), and nitrogenase complex. However, the genomes of the four biocatalytic strains differ in size (**Table 2**). *C. ljungdahlii* harbors the largest genome (4.63 Mbp), followed by *C. coskatii* (4.51 Mbp), *C. ragsdalei* (4.41 Mbp), and *C. autoethanogenum* (4.35 Mbp). The genome of *C. autoethanogenum* is 6.4% (0.28 Mbp) smaller than the one of *C. ljungdahlii*. The pan/core genome analysis is in accordance to the ANI analysis and underlines the conclusion that the strains *C. ljungdahlii, C. autoethanogenum, C. ragsdalei*, and *C. coskatii* belong to the same bacterial species. Due to the high similarity of their genome sequences the strains *C. autoethanogenum, C. ragsdalei*, and *C. coskatii* are subspecies of the validly described bacterial species *C. ljungdahlii.* The regions of differences contain mainly genes encoding parts of prophages, hypothetical proteins, methyltransferases, and transporters (**Figure 4**). Interestingly, the genomes of *C. autoethanogenum* and *C. ragsdalei* lack the genes encoding a glycine reductase complex. Thus, these strains should not be able to reduce glycine (Andreesen, 2004). However, the glycine decarboxylase complex (Freudenberg and Andreesen, 1989) is present in the genome of all strains.

### Engineering Industrial Acetogenic Biocatalysts

Recently, Hoffmeister et al. (2016) showed that recombinant *Acetobacetium woodii* strains that carry the plasmids pJIR_act_P_*_thalA_* and pJIR_act_Ppta-ack_ harboring ASO (encoding enzymes responsible for acetone formation), indeed produce acetone. In contrast, recombinant *C. ljungdahlii* strains harboring the identical plasmids produce 2-propanol. *C. ljungdahlii* harbors a gene (CLJU_24860) encoding a NADPH-dependent primary-secondary alcohol dehydrogenase converting acetone in 2-propanol (Köpke et al., 2014). Previously, the functionality of an equal ASO construct was demonstrated by Banerjee et al. (2014) using a comparable recombinant *C. ljungdahlii* strain. However, acetone and not 2-propanol production was determined in culture supernatant using a GC (‘clarus 600’) device (Banerjee et al., 2014). This might be due to an analytical problem differentiating acetone and 2-propanol using a GC device. Depending on the column used and temperature profile applied, acetone and 2-propanol have the nearly the same retention time (‘clarus 600,’ Porapak column). Acetone and 2-propanol are different bulk chemicals, which have different further applications. The largest applications for acetone are as a solvent and as an intermediate in the synthesis of bisphenol A, methyl methacrylate, and aldol chemicals (Weber et al., 2014). 2-Propanol is used primarily as a solvent in inks and surfactants. Further applications include its uses as an antiseptic alcohol, as a reaction solvent for cellulose carboxymethyl ether, in the production of cosmetic base materials and pesticide carriers, and for removal of water from gasoline tanks in cars (Papa, 2011). Banerjee et al. (2014) used a lactose-inducible expression system to control ASO construct, CO as substrate in uncontrolled batch experiments, and achieved up to 15 mM of 2-propanol. Thus, the ASO under control of the lactose-inducible promoter, P*_bgaL_* (Hartman et al., 2010), clearly outperformed the ASO controlled by promoters P*_thlA_* and P*_pta-ack_*, which were used in this study. However, P*_thlA_* was previously used in *C. ljungdahlii* to express the *adhE2* gene under heterotrophic growth conditions (Leang et al., 2013). It is noteworthy that under autotrophic growth conditions the strain *C. ljungdahlii* [pJIR_act_P_*_thlA_*] was not able to produce 2-propanol, whereas under heterotrophic conditions up to 5 mM were produced. Ueki et al. (2014) used a putative promoter region (550 bp) of *pta* gene to express eight genes of butyrate pathway. In this study, *pta-ack* promoter was used as determined by primer extension experiments (Hoffmeister et al., 2016). Nevertheless, results indicate that P*_pta-ack_* is stronger then P*_thlA_* since the *C. ljungdahlii* [pJIR_act_P_*_pta-ack_*] produced at least small amounts of 2-propanol (1.4 mM ± 0.5) under autotrophic growth conditions.

A further option to optimize the acetone/2-propanol production using acetogenic bacteria was recently discussed by Hoffmeister et al. (2016). The recombinant strain *A. woodii* [pMTL84151_actthlA] was used in a CSTR-bioreactor, and it was questioned whether the high Km value of the CoA transferase for acetate (1,200 mM) has an impact on acetone production. A change in fermentation technique led to an optimized acetone productivity of strain *A. woodii* [pMTL84151_actthlA] (1.2 mg L^-1^ h^-1^ in uncontrolled bottle fermentation, up to 26.4 mg L^-1^ h^-1^ in controlled continuous gas fermentations using a CSTR) (Hoffmeister et al., 2016).

The ClosTron^TM^ system (Heap et al., 2010) was used to construct an integration mutant of *C. ljungdahlii* [*adhE1*::intron]. The respective strain carries an inactivated *adhE1* gene due to insertion of the intron from plasmid pMTL007C-E2_adhE1::intron. *C. ljungdahlii* cells harboring the plasmid pMTL007C-E2_adhE1::intron were streaked onto solid growth medium supplemented with clarithromycin, and clones were readily isolated. The *C. ljungdahlii adhE1* integration mutant grew on syngas as the WT strain but showed an increased acetate:ethanol ratio (7:1) compared to WT strain (ratio 2:1) (see **Table 3**, **Figure 7**). Leang et al. (2013) constructed mutant alleles to disrupt *adhE1, adhE2*, or both by replacing the respective coding regions with the gene *ermC.* The ClosTron^TM^ system is based on the mobile group II intron from the *ltrB* gene of *Lactococcus lactis* (Ll.ltrB) that mediates the insertion of the gene *ermC* at a specific site of the target gene. Both *C. ljungdahlii adhE1* mutant strains showed impaired ability to produce ethanol. Although gene deletion systems for *C. ljungdahlii* were previously presented (Leang et al., 2013; Ueki et al., 2014), the ClosTron^TM^ system offers several advantages. Design and construction of the ClosTron^TM^ plasmid is quickly completed online^3^ without laboratory work. Within 2 or 3 weeks, the required plasmid is delivered by the company DNA2.0 (Menlo Park, CA, USA). Plasmid transfer in competent *C. ljungdahlii* cells is carried out using a standard method (Leang et al., 2013). Finally, mutant isolation simply requires recombinant cells to be transferred to growth medium supplemented with clarithromycin or lincomycin (Heap et al., 2010). The standard plasmid pMTL007C-E2 of the ClosTron^TM^ system carries the origin of replication (ori) pCB102 from *C. butyricum*. This ori is also functional in *A. woodii* (Hoffmeister et al., 2016). Therefore, it is likely that the ClosTron^TM^ system can also be applied in *A. woodii* to construct the mutant of interest.

### Glossary for Genome Analysis

**ANI-analysis**, average nucleotide identity analysis **COG**, The Clusters of Orthologous Groups (COGs) of proteins were computed by aligning the protein sequences of complete genomes. Each cluster comprises proteins or groups of paralogs from at least three lineages. The current COG database contains both prokaryotic clusters (COGs) and eukaryotic clusters (KOGs) (Galperin et al., 2014) **core genome**, genes present in all strains **dispensable genome**, genes present in two or more strains ***E*-value**, (Expect value) a parameter that describes the number of hits that can be “expected” to find by chance when searching a database. A low *E*-value (close to zero) indicates a significant match. **Locus tag**, numerical identifier of a gene in genome sequence **orthologous genes (orthologs)**, Copies of a single gene in two or more strains encoding a protein having the same function. **Pan genome** includes core genome, dispensable genome (OGs shared by at least two genomes) and genome specific OGs (singletons) **paralogs**, A pair of genes that derives from the same ancestral gene and now resides at different locations within the same genome. **Specific genome**, specific genes that occur only in a single strain.

## Author Contributions

FB conducted experiments, processed samples and drafted the manuscript. AP performed sequencing, annotation, depositing of genomes, and compiled comparative analyses of genome sequences. SH constructed plasmids. CE, SL, processed samples created figures, and wrote content of manuscript. TH conducted experiments and processed samples. RD and PD made interpretation of findings. PD supervised the workflow. All authors read, reviewed and approved the final manuscript.

## Conflict of Interest Statement

The authors declare that the research was conducted in the absence of any commercial or financial relationships that could be construed as a potential conflict of interest.

## Footnotes

1 http://www.bacterio.net/validationlists.html

2 http://clostron.com/clostron2.php

3 http://clostron.com

## Supplementary Material

FIGURE S1 **Growth profiles of C. *ljungdahlii* (A), C. *autoethanogenum* (B), C. *ragsdalei* (C), and C. *coskatii* (D) cultivated in 1 L Müller-Krempel bottles on syngas in 100 mL Tanner mod.** Medium in an uncontrolled batch fermentation: diamond, OD_600_; triangle, acetate; square, ethanol; circle, 2,3-butanediol. Error bars show standard deviations.

FIGURE S2 **Phylogenetic tree based on amino acid sequences of alcohol dehydrogenase.** COG1062 (FrmA), Zn-dependent alcohol dehydrogenase [general function prediction only]; COG1063 (Tdh), threonine dehydrogenase or related Zn-dependent dehydrogenase [amino acid transport and metabolism, general function prediction only]; COG1454 (EutG), alcohol dehydrogenase class IV [energy production and conversion]; COG1979 (YqhD), alcohol dehydrogenase YqhD, Fe-dependent ADH family [energy production and conversion]; unclassified COG, alcohol dehydrogenase; DHQ-FeADH superfamily, dehydroquinate synthase-like (DHQ-like) and iron-containing alcohol dehydrogenase (Fe-ADH) (cl02872). Designations of alcohol dehydrogenases correspond to respective names given in genome sequences.

TABLE S1 **Orthologous genes (orthologs) among genome sequences of *C. ljungdahlii, C. autoethanogenum, C. ragsdalei*, and *C. coskatii* identified using the program Proteinortho version 4.26 listed in an excel file**.

TABLE S2 **Autotrophic growth characteristics of clostridial wild type (WT) strains in uncontrolled batch cultivation**.
