# Supplementary material for: Industrial Acetogenic Biocatalysts: A Comparative Metabolic and Genomic Analysis
Source: Front Microbiol. 2016 Jul 7;7:1036. doi: 10.3389/fmicb.2016.01036 (PMC4935695; doi:10.3389/fmicb.2016.01036)
Supplement: FIGURE S1 — Growth profiles of C. ljungdahlii (A), C. autoethanogenum (B), C. ragsdalei (C), and C. coskatii (D) cultivated in 1 L Müller-Krempel bottles on syngas in 100 mL Tanner mod. Medium in an uncontrolled batch fermentation: diamond, OD600; triangle, acetate; square, ethanol; circle, 2,3-butanediol. Error bars show standard deviations. [file Image_1.PDF]

Supplementary material

The supplementary material for this article can be found online at:

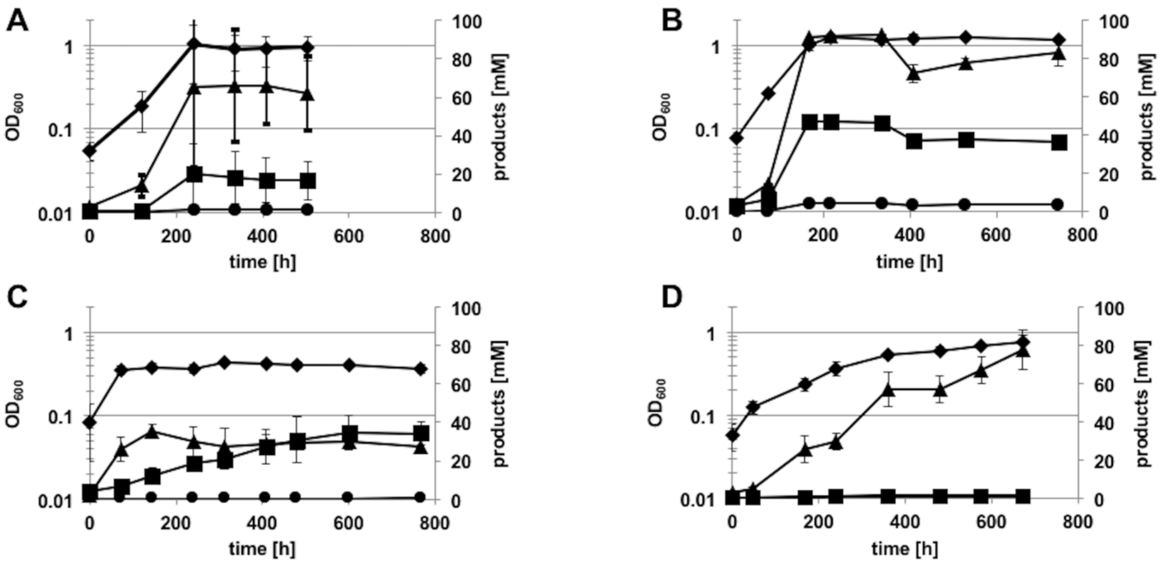

Figure S1. Growth profiles of *C. ljungdahlii* (A), *C. autoethanogenum* (B), *C. ragsdalei* (C), and *C. coskatii* (D) cultivated in 1 L Müller-Krempel bottles on syngas in 100 mL Tanner mod. Medium in an uncontrolled batch fermentation: diamond, OD_{600}; triangle, acetate; square, ethanol; circle, 2,3-butanediol. Error bars show standard deviations.
