# Supplementary material for: Industrial Acetogenic Biocatalysts: A Comparative Metabolic and Genomic Analysis
Source: Front Microbiol. 2016 Jul 7;7:1036. doi: 10.3389/fmicb.2016.01036 (PMC4935695; doi:10.3389/fmicb.2016.01036)
Supplement: FIGURE S2 — Phylogenetic tree based on amino acid sequences of alcohol dehydrogenase. COG1062 (FrmA), Zn-dependent alcohol dehydrogenase [general function prediction only]; COG1063 (Tdh), threonine dehydrogenase or related Zn-dependent dehydrogenase [amino acid transport and metabolism, general function prediction only]; COG1454 (EutG), alcohol dehydrogenase class IV [energy production and conversion]; COG1979 (YqhD), alcohol dehydrogenase YqhD, Fe-dependent ADH family [energy production and conversion]; unclassified COG, alcohol dehydrogenase; DHQ-FeADH superfamily, dehydroquinate synthase-like (DHQ-like) and iron-containing alcohol dehydrogenase (Fe-ADH) (cl02872). Designations of alcohol dehydrogenases correspond to respective names given in genome sequences. [file Image_2.PDF]

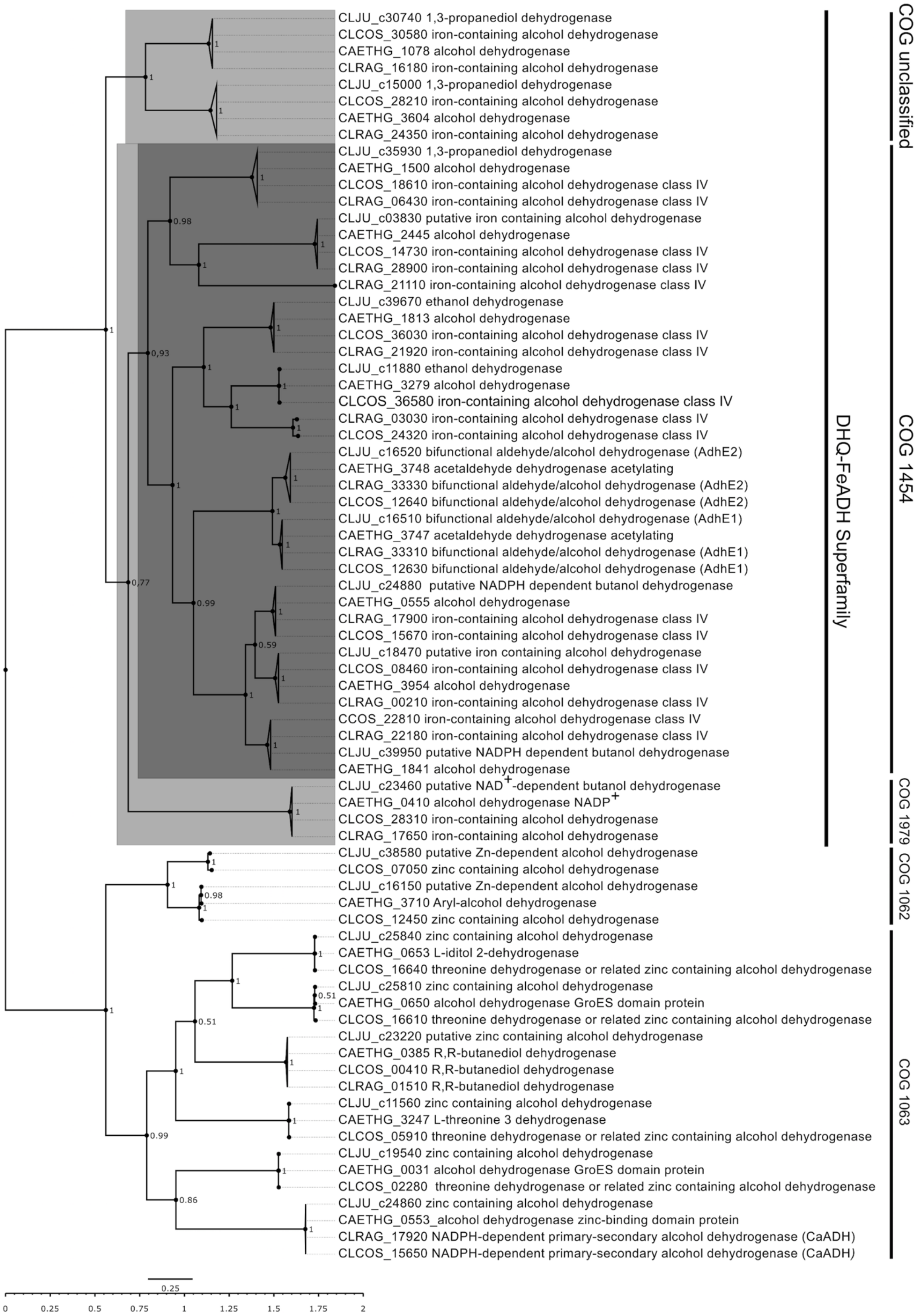
CLJU_c30740 1,3-propanediol dehydrogenase
CLCOS_30580 iron-containing alcohol dehydrogenase
CAETHG_1078 alcohol dehydrogenase
CLRAG_16180 iron-containing alcohol dehydrogenase
CLJU_c15000 1,3-propanediol dehydrogenase
CLCOS_28210 iron-containing alcohol dehydrogenase
CAETHG_3604 alcohol dehydrogenase
CLRAG_24350 iron-containing alcohol dehydrogenase
CLJU_c35930 1,3-propanediol dehydrogenase
CAETHG_1500 alcohol dehydrogenase
CLCOS_18610 iron-containing alcohol dehydrogenase class IV
CLRAG_06430 iron-containing alcohol dehydrogenase class IV
CLJU_c03830 putative iron containing alcohol dehydrogenase
CAETHG_2445 alcohol dehydrogenase
CLCOS_14730 iron-containing alcohol dehydrogenase class IV
CLRAG_28900 iron-containing alcohol dehydrogenase class IV
CLRAG_21110 iron-containing alcohol dehydrogenase class IV
CLJU_c39670 ethanol dehydrogenase
CAETHG_1813 alcohol dehydrogenase
CLCOS_36030 iron-containing alcohol dehydrogenase class IV
CLRAG_21920 iron-containing alcohol dehydrogenase class IV
CLJU_c11880 ethanol dehydrogenase
CAETHG_3279 alcohol dehydrogenase
CLCOS_36580 iron-containing alcohol dehydrogenase class IV
CLRAG_03030 iron-containing alcohol dehydrogenase class IV
CLCOS_24320 iron-containing alcohol dehydrogenase class IV
CLJU_c16520 bifunctional aldehyde/alcohol dehydrogenase (AdhE2)
CAETHG_3748 acetaldehyde dehydrogenase acetylating
CLRAG_33330 bifunctional aldehyde/alcohol dehydrogenase (AdhE2)
CLCOS_12640 bifunctional aldehyde/alcohol dehydrogenase (AdhE2)
CLJU_c16510 bifunctional aldehyde/alcohol dehydrogenase (AdhE1)
CAETHG_3747 acetaldehyde dehydrogenase acetylating
CLRAG_33310 bifunctional aldehyde/alcohol dehydrogenase (AdhE1)
CLCOS_12630 bifunctional aldehyde/alcohol dehydrogenase (AdhE1)
CLJU_c24880 putative NADPH dependent butanol dehydrogenase
CAETHG_0555 alcohol dehydrogenase
CLRAG_17900 iron-containing alcohol dehydrogenase class IV
CLCOS_15670 iron-containing alcohol dehydrogenase class IV
CLJU_c18470 putative iron containing alcohol dehydrogenase
CLCOS_08460 iron-containing alcohol dehydrogenase class IV
CAETHG_3954 alcohol dehydrogenase
CLRAG_00210 iron-containing alcohol dehydrogenase class IV
CCOS_22810 iron-containing alcohol dehydrogenase class IV
CLRAG_22180 iron-containing alcohol dehydrogenase class IV
CLJU_c39950 putative NADPH dependent butanol dehydrogenase
CAETHG_1841 alcohol dehydrogenase
CLJU_c23460 putative NAD+-dependent butanol dehydrogenase
CAETHG_0410 alcohol dehydrogenase NADP+
CLCOS_28310 iron-containing alcohol dehydrogenase
CLRAG_17650 iron-containing alcohol dehydrogenase
CLJU_c38580 putative Zn-dependent alcohol dehydrogenase
CLCOS_07050 zinc containing alcohol dehydrogenase
CLJU_c16150 putative Zn-dependent alcohol dehydrogenase
CAETHG_3710 Aryl-alcohol dehydrogenase
CLCOS_12450 zinc containing alcohol dehydrogenase
CLJU_c25840 zinc containing alcohol dehydrogenase
CAETHG_0653 L-iditol 2-dehydrogenase
CLCOS_16640 threonine dehydrogenase or related zinc containing alcohol dehydrogenase
CLJU_c25810 zinc containing alcohol dehydrogenase
CAETHG_0650 alcohol dehydrogenase GroES domain protein
CLCOS_16610 threonine dehydrogenase or related zinc containing alcohol dehydrogenase
CLJU_c23220 putative zinc containing alcohol dehydrogenase
CAETHG_0385 R,R-butanediol dehydrogenase
CLCOS_00410 R,R-butanediol dehydrogenase
CLRAG_01510 R,R-butanediol dehydrogenase
CLJU_c11560 zinc containing alcohol dehydrogenase
CAETHG_3247 L-threonine 3 dehydrogenase
CLCOS_05910 threonine dehydrogenase or related zinc containing alcohol dehydrogenase
CLJU_c19540 zinc containing alcohol dehydrogenase
CAETHG_0031 alcohol dehydrogenase GroES domain protein
CLCOS_02280 threonine dehydrogenase or related zinc containing alcohol dehydrogenase
CLJU_c24860 zinc containing alcohol dehydrogenase
CAETHG_0553_alcohol dehydrogenase zinc-binding domain protein
CLRAG_17920 NADPH-dependent primary-secondary alcohol dehydrogenase (CaADH)
CLCOS_15650 NADPH-dependent primary-secondary alcohol dehydrogenase (CaADH)
DHQ-FeADH Superfamily
COG unclassified
COG 1454
COG 1979
COG 1062
COG 1063
0.98
0,93
0,77
0.99
0.59
0.51
0.86
0.25
0 0.25 0.5 0.75 1 1.25 1.5 1.75 2

Figure S2 (Data Sheet 2). Phylogenetic tree based on amino acid sequences of alcohol dehydrogenase. COG1062 (FrmA), Zn-dependent alcohol dehydrogenase [general function prediction only]; COG1063 (Tdh), threonine dehydrogenase or related Zn-dependent dehydrogenase [amino acid transport and metabolism, general function prediction only]; COG1454 (EutG), alcohol dehydrogenase class IV [energy production and conversion]; COG1979 (YqhD), alcohol dehydrogenase YqhD, Fe-dependent ADH family [energy production and conversion]; unclassified COG, alcohol dehydrogenase; DHQ-FeADH superfamily, dehydroquinate synthase-like (DHQ-like) and iron-containing alcohol dehydrogenase (Fe-ADH) (cl02872). Designations of alcohol dehydrogenases correspond to respective names given in genome sequences.
