# Supplementary material for: Industrial Acetogenic Biocatalysts: A Comparative Metabolic and Genomic Analysis
Source: Front Microbiol. 2016 Jul 7;7:1036. doi: 10.3389/fmicb.2016.01036 (PMC4935695; doi:10.3389/fmicb.2016.01036)
Supplement: TABLE S2 — Autotrophic growth characteristics of clostridial wild type (WT) strains in uncontrolled batch cultivation. [file Table_2.DOCX]

Table S2. Autotrophic growth characteristics of clostridial wild type (WT) strains in uncontrolled batch cultivation.

| Strain (WT) | Max. OD_600_ | Acetate  [mM] | Ethanol  [mM] | 2,3-Butanediol  [mM] |
| --- | --- | --- | --- | --- |
| *C. ljungdahlii* | 1.0 ± 0.3 | 62.0 ± 19.0 | 16.5 ± 10.2 | 1.4 ± 0.7 |
| *C. autoethanogenum* | 1.27 ± 0.1 | 77.7 ± 2.7 | 37.7 ± 0.9 | 3.7 ± 0.3 |
| *C. ragsdalei* | 0.43 ± 0.01 | 30.1 ± 4.5 | 34.7 ± 8.7 | Not detected ^1^ |
| *C. coskatii* | 0.76 ± 0.17 | 66.9 ±7.1 | 1.4 ± 0.3 | 1.1 ± 0.2 |

Data represent mean values after 530 h incubation; ±, standard deviations of biological triplicates; ^1^, limit of detection 0.7 mM.
